# Supplementary material for: Lacticaseibacillus rhamnosus GG Versus Placebo for Eradication of Vancomycin-Resistant Enterococcus faecium in Intestinal Carriers: A Systematic Review and Meta-Analysis
Source: Microorganisms. 2023 Nov 19;11(11):2804. doi: 10.3390/microorganisms11112804 (PMC10673360; doi:10.3390/microorganisms11112804)
Supplement: Supplementary file 1 [file microorganisms-11-02804-s001.zip › microorganisms-2663761-supplementary.pdf]

Supplementary document 1

21-08-23: History and search details from PubMed, Embase, and Cochrane Library

PubMed Search:

Search	Actions	Details	Query	Results	Time
#5			Search: **((("Lacticaseibacillus rhamnosus"[Mesh]) OR "Probiotics"[Mesh]) OR (("Lacticaseibacillus rhamnosus"[Text word] OR Probiotic*[Text word] OR "lactobacillus rhamnosus"[Text Word]))) AND (((("Vancomycin Resistance"[Mesh]) OR "Vancomycin-Resistant Enterococci"[Mesh]) OR "Enterococcus faecium"[Mesh]) OR ((Vancomycin resistan*[Text Word] OR Antibiotic resistan*[Text Word] OR "Enterococcus faecium"[Text Word] OR "E faecium"[Text Word] OR Enterococci[Text Word]))) NOT ("Animals"[Mesh] NOT "Humans"[Mesh])** Filters: **Danish, English, Norwegian, Swedish**	1,634	07:09:49
#1			Search: **((("Lacticaseibacillus rhamnosus"[Mesh]) OR "Probiotics"[Mesh]) OR (("Lacticaseibacillus rhamnosus"[Text word] OR Probiotic*[Text word] OR "lactobacillus rhamnosus"[Text Word]))) AND (((("Vancomycin Resistance"[Mesh]) OR "Vancomycin-Resistant Enterococci"[Mesh]) OR "Enterococcus faecium"[Mesh]) OR ((Vancomycin resistan*[Text Word] OR Antibiotic resistan*[Text Word] OR "Enterococcus faecium"[Text Word] OR "E faecium"[Text Word] OR Enterococci[Text Word]))) NOT ("Animals"[Mesh] NOT "Humans"[Mesh])**		

Embase Search:

1. lactobacillus rhamnosus/
2. exp probiotic agent/
3. ("Lacticaseibacillus rhamnosus" or Probiotic* or "lactobacillus rhamnosus").mp. [mp=title, abstract, heading word, drug trade name, original title, device manufacturer, drug manufacturer, device trade name, keyword heading word, floating subheading word, candidate term word]
4. 1 or 2 or 3
5. vancomycin resistance/
6. vancomycin resistant enterococcus/
7. enterococcus faecium/
8. (Vancomycin resistan* or Antibiotic resistan* or "Enterococcus faecium" or "E faecium" or Enterococci).mp. [mp=title, abstract, heading word, drug trade name, original title, device manufacturer, drug manufacturer, device trade name, keyword heading word, floating subheading word, candidate term word]
9. 5 or 6 or 7 or 8
10. 4 and 9
11. exp animal/ not exp Human/
12. 10 not 11
13. limit 12 to (conference abstract or conference paper or "conference review" or editorial or letter or note)
14. 12 not 13
15. limit 14 to (danish or english or norwegian or swedish)

Cochrane Search:

1. MeSH descriptor: Lacticaseibacillus rhamnosus
2. MeSH descriptor: Probiotics
3. Lacticaseibacillus rhamnosus OR Probiotic OR Lactobacillus rhamnosus
4. #1 OR #2 OR#3
5. MeSH descriptor: Vancomycin Resistance
6. MeSH descriptor: Vancomycin Resistant Enterococci
7. MeSH descriptor: Enterococcus Faecium
8. Vancomycin Resistant OR Antibiotic Resistant OR Enterococcus Faecium OR E.faecium
9. #5 OR #6 OR #7 OR #8
10. #4 AND #9

Supplementary document 2

Criteria	Yes	No	Other (CD, NR, NA)*
1. Was the study described as randomized, a randomized trial, a randomized clinical trial, or an RCT?			
2. Was the method of randomization adequate (i.e., use of randomly generated assignment)?			
3. Was the treatment allocation concealed (so that assignments could not be predicted)?			
4. Were study participants and providers blinded to treatment group assignment?			
5. Were the people assessing the outcomes blinded to the participants' group assignments?			
6. Were the groups similar at baseline on important characteristics that could affect outcomes (e.g., demographics, risk factors, co-morbid conditions)?			

Criteria	Yes	No	Other (CD, NR, NA)*
7. Was the overall drop-out rate from the study at endpoint 20% or lower of the number allocated to treatment?			
8. Was the differential drop-out rate (between treatment groups) at endpoint 15 percentage points or lower?			
9. Was there high adherence to the intervention protocols for each treatment group?			
10. Were other interventions avoided or similar in the groups (e.g., similar background treatments)?			
11. Were outcomes assessed using valid and reliable measures, implemented consistently across all study participants?			
12. Did the authors report that the sample size was sufficiently large to be able to detect a difference in the main outcome between groups with at least 80% power?			

Criteria	Yes	No	Other (CD, NR, NA)*
13. Were outcomes reported or subgroups analyzed prespecified (i.e., identified before analyses were conducted)?			
14. Were all randomized participants analyzed in the group to which they were originally assigned, i.e., did they use an intention-to-treat analysis?			

*CD, cannot determine; NA, not applicable; NR, not reported

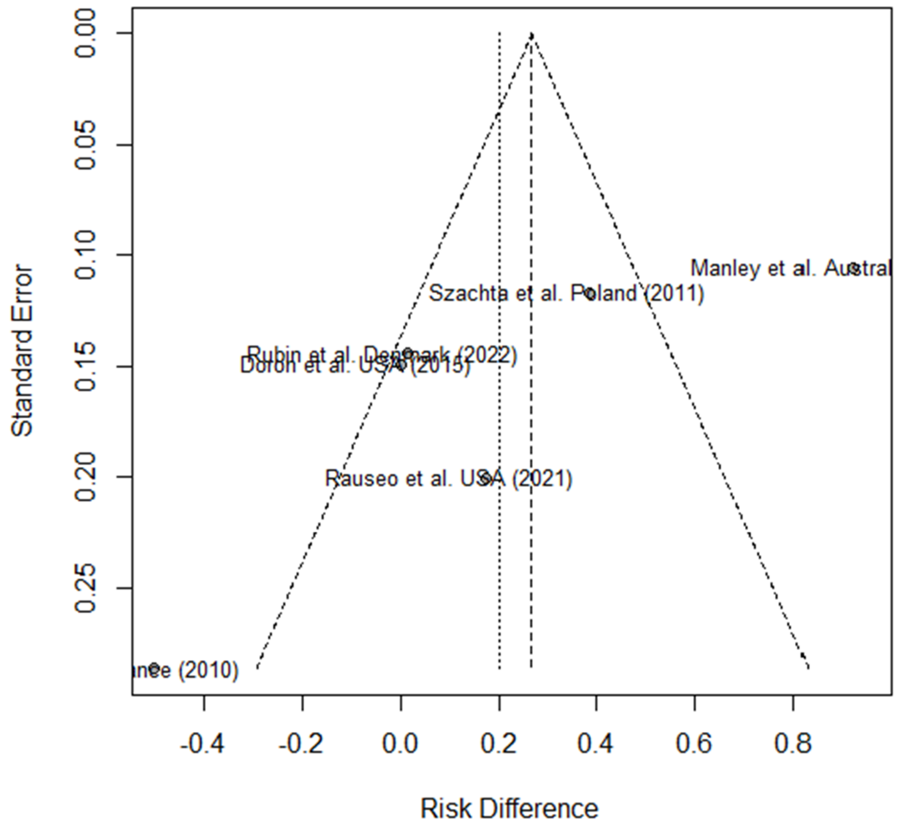

Figure S1 shows the funnel plot, where two of the six studies appear outside the plot.
